# Diagnostic Accuracy of Smartphone-Based Audiometry for Hearing Loss Detection: Meta-analysis

**DOI:** 10.2196/28378

**Published:** 2021-09-10

**Authors:** Chih-Hao Chen, Heng-Yu Haley Lin, Mao-Che Wang, Yuan-Chia Chu, Chun-Yu Chang, Chii-Yuan Huang, Yen-Fu Cheng

**Affiliations:** 1 Department of Otolaryngology-Head and Neck Surgery Taipei Veterans General Hospital Taiwan Taipei City Taiwan; 2 Department of Medical Education Taipei Veterans General Hospital Taipei City Taiwan; 3 Faculty of Medicine National Yang Ming Chiao Tung University Taipei City Taiwan; 4 Institute of Hospital and Health Care Administration National Yang Ming Chiao Tung University Taipei City Taiwan; 5 Information Management Office Taipei Veterans General Hospital Taipei City Taiwan; 6 Medical AI Development Center Taipei Veterans General Hospital Taipei City Taiwan; 7 Department of Information Management National Taipei University of Nursing and Health Sciences Taipei City Taiwan; 8 Department of Anesthesiology Taipei Tzu Chi Hospital Buddhist Tzu Chi Medical Foundation New Taipei City Taiwan; 9 Department of Medical Research Taipei Veterans General Hospital Taipei City Taiwan; 10 Institute of Brain Science National Yang Ming Chiao Tung University Taipei City Taiwan

**Keywords:** audiometry, hearing loss, hearing test, mhealth, mobile health, digital health, meta-analysis, mobile phone, smartphone diagnostic test accuracy

## Abstract

**Background:**

Hearing loss is one of the most common disabilities worldwide and affects both individual and public health. Pure tone audiometry (PTA) is the gold standard for hearing assessment, but it is often not available in many settings, given its high cost and demand for human resources. Smartphone-based audiometry may be equally effective and can improve access to adequate hearing evaluations.

**Objective:**

The aim of this systematic review is to synthesize the current evidence of the role of smartphone-based audiometry in hearing assessments and further explore the factors that influence its diagnostic accuracy.

**Methods:**

Five databases—PubMed, Embase, Cochrane Library, Web of Science, and Scopus—were queried to identify original studies that examined the diagnostic accuracy of hearing loss measurement using smartphone-based devices with conventional PTA as a reference test. A bivariate random-effects meta-analysis was performed to estimate the pooled sensitivity and specificity. The factors associated with diagnostic accuracy were identified using a bivariate meta-regression model. Study quality was assessed using the Quality Assessment of Diagnostic Accuracy Studies-2 tool.

**Results:**

In all, 25 studies with a total of 4470 patients were included in the meta-analysis. The overall sensitivity, specificity, and area under the receiver operating characteristic curve for smartphone-based audiometry were 89% (95% CI 83%-93%), 93% (95% CI 87%-97%), and 0.96 (95% CI 0.93-0.97), respectively; the corresponding values for the smartphone-based speech recognition test were 91% (95% CI 86%-94%), 88% (95% CI 75%-94%), and 0.93 (95% CI 0.90-0.95), respectively. Meta-regression analysis revealed that patient age, equipment used, and the presence of soundproof booths were significantly related to diagnostic accuracy.

**Conclusions:**

We have presented comprehensive evidence regarding the effectiveness of smartphone-based tests in diagnosing hearing loss. Smartphone-based audiometry may serve as an accurate and accessible approach to hearing evaluations, especially in settings where conventional PTA is unavailable.

## Introduction

### Background

Hearing loss is one of the most common disabilities affecting both individual and public health. Hearing loss has been linked to multiple physical [1,2], cognitive [3,4], and psychosocial [5,6] outcomes and is associated with problematic health care use and higher medical expenses [7]. According to previous studies and World Health Organization estimates, more than 5% of the world’s population is affected by hearing impairment, especially older adults aged above 65 years [8-10]. Notably, the prevalence of hearing loss is 50% higher in low-income countries [11]. Within the disease spectrum of hearing impairment, a considerable number of cases, such as those involving idiopathic sudden sensorineural hearing loss (SSNHL) and noise-induced hearing loss, are preventable and can be treated effectively and in a timely manner [12-14].

Pure tone audiometry (PTA) is the gold standard for current hearing assessment batteries [15]. However, this measurement is often unavailable, given its demanding nature with regard to equipment, certified personnel, space, and expenses, particularly in settings such as primary care practices, urgent care, and in low- and middle-income countries [16-18]. As hearing loss has been identified as the single largest potentially modifiable risk factor for dementia in midlife [19] and most patients with hearing impairment can benefit from timely interventions, a more accessible and equally accurate approach to hearing assessment is warranted. Great efforts have been made to create more cost-effective devices and automate audiologic examinations, resulting in the rapid development of smartphone audiometry. Because of the universal availability of mobile technology and cellular networks, smartphone-based hearing tests may provide an adequate assessment of hearing as an alternative to conventional PTA and assist large-scale hearing screening [16,20,21].

### Objective

A considerable number of smartphone apps have been introduced for hearing screening [22,23], evaluation [24-26], and even rehabilitation and care [27,28] in recent years, and previous research has compared the performance of these apps with standard audiometry [21,29]. However, these studies were heterogeneous in terms of study design, use of equipment, and baseline characteristics of the participants, which resulted in inconsistent data on the diagnostic performance of smartphone audiometry. The aim of this study is to synthesize the most updated and comprehensive evidence of the diagnostic value of smartphone-based hearing assessments for hearing loss. We performed a meta-analysis with meta-regression to summarize the diagnostic accuracy of smartphone audiometry and investigated the factors affecting the test results. We aim to provide more definitive evidence of the utility of smartphone audiometry in clinical application in the future.

## Methods

### Study Design

This meta-analysis followed the PRISMA (Preferred Reporting Items for Systematic Reviews and Meta-analyses) Diagnostic Test Accuracy Studies statement [30].

### Search Strategy

In all, five databases—PubMed, Embase, Cochrane Library, Web of Science and Scopus—were searched from inception through January 15, 2021, by 2 authors (CHC and HYHL). The Boolean operator *OR* was used to cover similar concepts, whereas *AND* was used to intersect different concepts. We used a combination of Medical Subject Headings and text words to create three subsets of citations: the first included studies on hearing loss (*hearing loss*, *hypoacusis*, and *hearing impairment*), the second included studies on smartphones (*smartphone*, *cellular phone*, *mobile*, and *mobile phone*), and the third included studies on the concept of use (*diagnosis*, *audiometry*, and *self-examination*). The detailed search strategy is presented in Multimedia Appendix 1. The identified citations were imported into the reference software and screened by title, abstract, and keyword. Potentially eligible records were then subjected to a full-text review.

### Eligibility Criteria

The included studies were selected based on the following criteria: (1) PTA was used as a reference test, (2) audiometry was used on smart devices (ie, PTA and speech recognition audiometry) as an index test, and (3) adequate information was reported on diagnostic accuracy (ie, prevalence, sensitivity, and specificity) to quantify the effect estimates for meta-analysis. Studies with outcomes that did not relate to the diagnostic accuracy of the index test or did not provide enough information for meta-analysis were excluded. We did not exclude studies based on country, language, or publication date.

### Study Selection and Data Extraction

All studies were fully reviewed and selected by 2 authors (CHC and HYHL). If there were any disagreements in the study selection, they were resolved by a third author (YFC) through consensus or discussion. The extracted data included the author’s name, publication year, country, test setting, number of patients, mean age of the study population, operating system of the smart device, equipment used during the examination, and use of a soundproof booth. The disease population was defined as comprising patients with abnormal reference test results in each study. The quantitative data were either extracted directly from raw data or converted from the diagnostic parameters (ie, sensitivity, specificity, and prevalence) in each study to construct standard diagnostic test 2×2 tables containing true-positive, false-positive, false-negative, and true-negative samples for the index text.

### Study Quality Assessment

The quality of the included studies was assessed by 2 authors (CHC and HYHL) using the Quality Assessment of Diagnostic Accuracy Studies-2 tool. A third reviewer (YFC) resolved disagreements regarding the methodological quality through consensus or discussion.

### Statistical Analysis

#### Overview

Sensitivity and specificity were calculated for each extracted data set. A negative correlation between sensitivity and specificity caused by different thresholds was observed; therefore, we adopted a bivariate random-effects model to estimate the pooled sensitivity and specificity of the index test and to account for the heterogeneity that commonly exists in meta-analyses of diagnostic accuracy tests [31]. The bivariate random-effects model assumes logit-transformed sensitivity and specificity as bivariable distributions, and it also considers the threshold effect, which is an indication of the trade-off phenomenon in most diagnostic accuracy tests because the threshold differs among studies [32]. To investigate the covariate among the index studies, bivariate meta-regression analysis was performed [33], one at a time. For the covariate effect on age, we divided the studies into child, elderly, and adult groups. People aged below 18 years were considered to be in the child group, whereas people aged above 65 years were considered to be in the elderly group based on the World Health Organization criteria [34]. First, we examined whether the covariate caused variance in the sensitivity and specificity measures. The following likelihood-ratio chi-square test was used to determine whether the covariate served as a significant variable by testing the hypothesis that these covariates do not explain variance in the logit-transformed pairs of sensitivity and specificity. To further illustrate the diagnostic accuracy and compare the discriminatory properties, we constructed hierarchical summary receiver operating characteristic curves for the overall result as well as the subgroup results identified by the meta-regression analysis by accounting for the correlation in the data through a hierarchical approach. To deal with zero observations in the 2×2 contingency tables, 0.5 was added to each cell to reduce the influence of small studies. We calculated 95% CIs on the basis of the binominal distribution of the truly positive and truly negative samples. Publication bias was examined using the Deeks funnel plot using the natural logarithm of the diagnostic odds ratio against 1/(effective sample size)^1/2^ to plot the asymmetry of the included studies. Effective sample size (ESS) was calculated by the number of examinees who were diseased (n1) and not diseased (n2) as:

ESS = (4n1 n2) / (n1 + n2) **(1)**

ESS considers that unequal numbers of individuals who are diseased and not diseased reduce the precision of test accuracy estimates [31,35]. A *P*<.10 for the regression tests suggests significant publication bias. Statistical analyses were conducted using Stata version 15.0 (StataCorp), with the midas and metandi commands. All statistical tests were two-sided, and *P*<.05 was considered statistically significant.

## Results

### Study Identification and Selection

**Figure 1:**
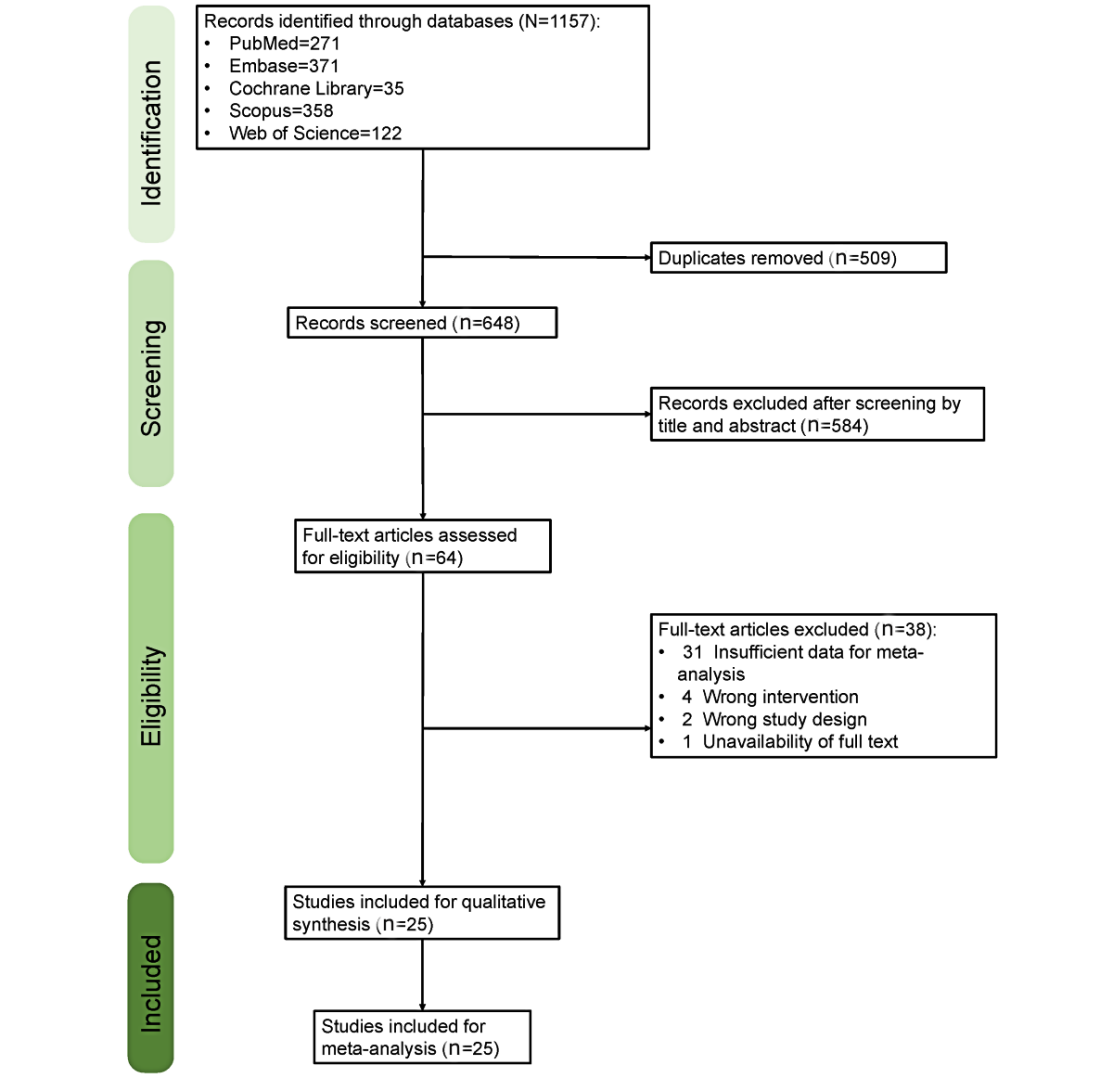
The PRISMA (Preferred Reporting Items for Systematic Reviews and Meta-analyses) ﬂow diagram.

A total of 1157 studies were identified through the databases. Of the 1157 studies, 648 (56%) remained in the preliminary search after the removal of 509 (44%) duplicates. Of the 648 studies, 584 (90.1%) were excluded after 2 authors (CHC and HYHL) screened the titles and abstracts; a total of 9.9% (64/648) of studies then underwent full-text review. Of the 64 studies, 39 (61%) were excluded because of the following reasons: insufficient data for meta-analysis, index tests not used, inappropriate study design, or unavailability of the full text. As a result, of the 64 studies, 25 (39%) studies with a total of 4470 patients were included in the meta-analysis. The detailed PRISMA ﬂow diagram is presented in Figure 1.

### Study Characteristics

Of the 25 studies, 21 were prospective [10,21,22,29,36-52], 1 was retrospective [53], and the remaining 3 studies did not report the study design [23,54,55]. In all, 20 studies used PTA as the index test [10,21,22,29,36-42,44-46,48-52,54], whereas the remaining 5 studies applied a speech recognition test (SRT) as the index test [23,43,47,53,55]. A total of 4 studies enrolled elderly participants [10,36,37,39], whereas 7 studies included children [21-23,38,41,49,55], and 13 studies enrolled adult participants [29,40,42-44,46,48-54]. The remaining study did not report the age of the study population [45]. In all, 15 studies operated audiometry through an iPhone (Apple Inc) operating system–based app [10,22,29,36,37,39,40,45-48,50,52,54,55], whereas the remaining 10 used an Android (Google LLC) operating system–based audiometry app [21,23,38,41-44,49,51,53]. A total of 15 studies used headphones for testing [10,21,23,38,41-47,49,50,52,54], 9 studies used earphones for the examination [22,29,36,37,39,40,48,53,55], and 1 study did not mention the equipment used [51]. In all, 6 studies conducted the examination in a soundproof booth [10,49-51,53,55], 18 studies did not use a soundproof booth to conduct the examination [21-23,29,36-47,52,54], and 1 study did not report whether the test was conducted in a soundproof booth [48]. A total of 4 studies [45,46,49,50] conducted the index test among different independent populations, yielding a total of 30 study groups for the analysis. Further information regarding the included study populations and statistics is presented in Multimedia Appendices 2 and 3.

### Quality and Risk-of-Bias Assessment

**Figure 2:**
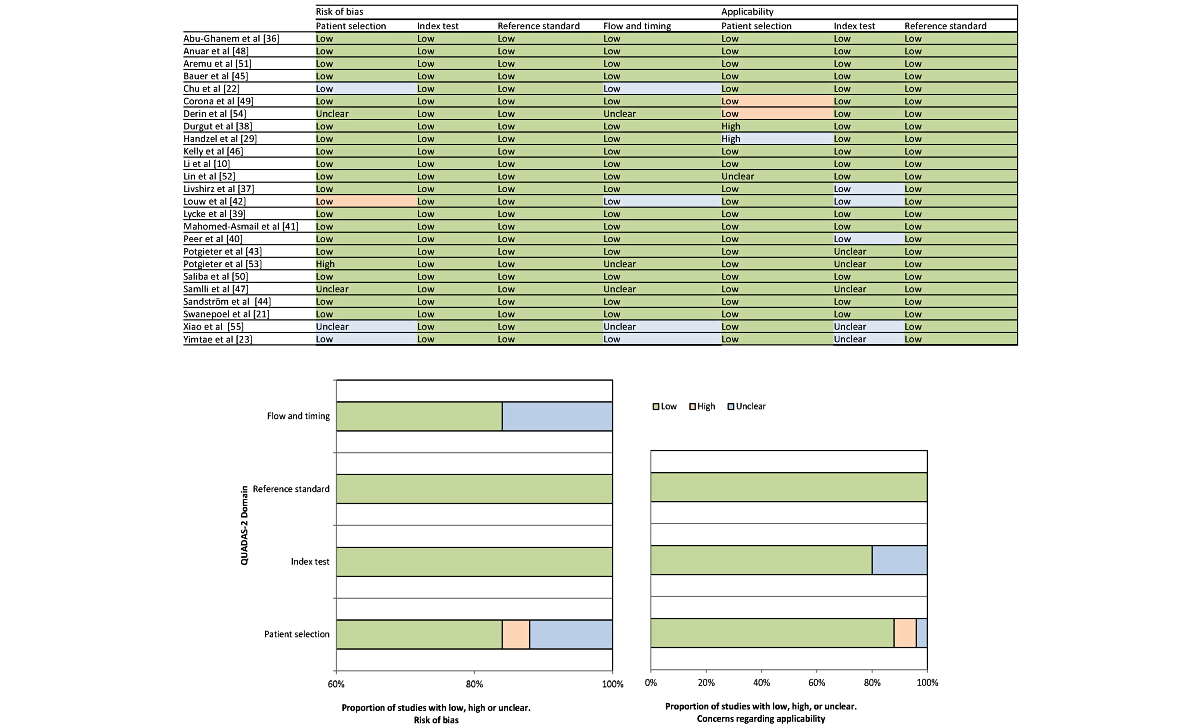
Quality assessment results based on the Quality Assessment of Diagnostic Accuracy Studies-2 (QUADAS-2) guidelines.

Quality Assessment of Diagnostic Accuracy Studies-2 scores were used to evaluate the quality of the included studies. Regarding the evaluation of the risk of bias, all the studies carried out index studies without knowing the results of the reference test in advance and set the threshold before testing. A total of 4 studies did not clearly describe the sequence between the index and reference tests [47,53-55]. Regarding the evaluation of applicability, 1 study enrolled patients with underlying otitis media [38], and another 2 studies included patients with SSNHL [29,52]. In all, 5 studies used unmarketed apps as index tests. A detailed assessment and an overall picture of the methodological quality of the included studies are presented in Figure 2.

### Overall Diagnostic Performance

**Figure 3:**
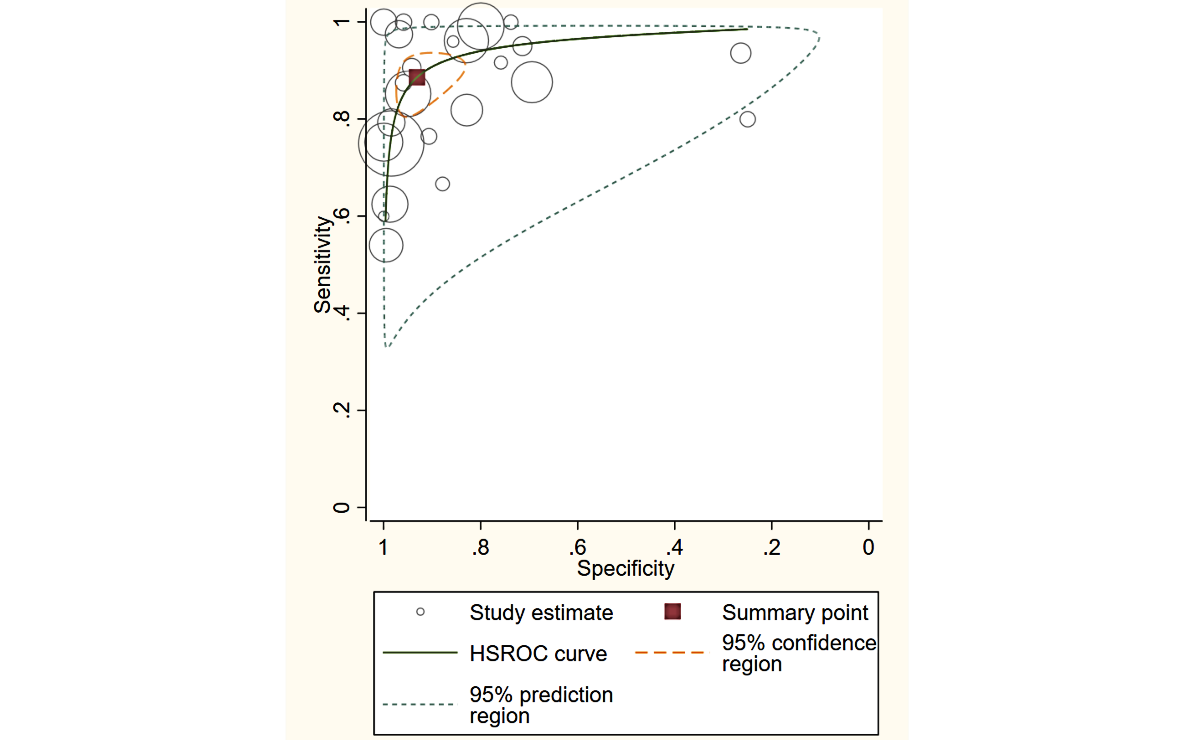
The HSROC for pure tone audiometry. HSROC: hierarchical summary receiver operating characteristic.

**Figure 4:**
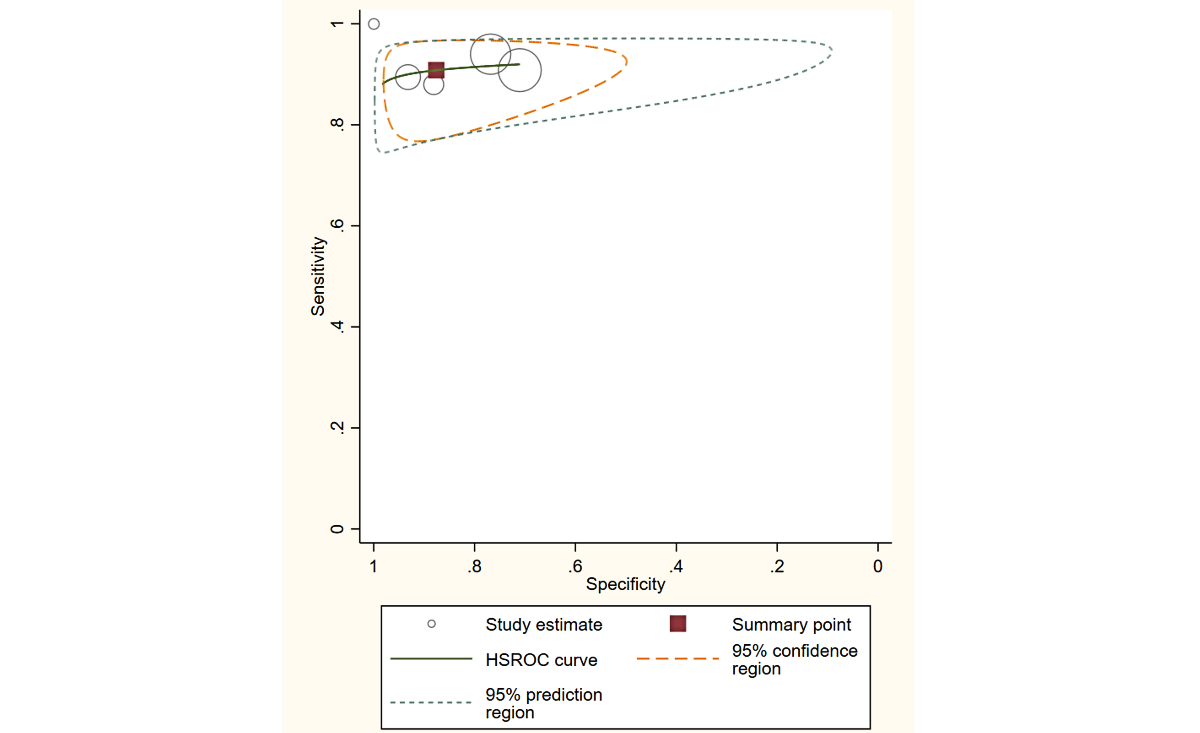
The HSROC for the speech recognition test. HSROC: hierarchical summary receiver operating characteristic.

Overall, the studies using a smartphone app with PTA showed a sensitivity of 89% (95% CI 83%-93%) and specificity of 93% (95% CI 87%-97%), whereas studies using an app involving SRT revealed a sensitivity of 91% (95% CI 86%-94%) and specificity of 88% (95% CI 75%-94%). The hierarchical summary receiver operating characteristic curves with summary points for both PTA and SRT are shown in Figures 3 and 4. The predicted values for the area under the receiver operating characteristic curve (AUC) for the PTA and SRT measures were 0.96 (95% CI 0.93-0.97) and 0.93 (95% CI 0.90-0.95), respectively.

### Meta-Regression and Subgroup Analysis

The bivariate meta-regression analysis showed a significant influence of the operating system on sensitivity (88% vs 89%). The likelihood-ratio chi-square test revealed that elderly group (*χ*^2^_1_=85.9; *P*<.001), child group (*χ*^2^_1_=62.9; *P*<.001), headphone use (*χ*^2^_1_=17.8; *P*<.001), and soundproof booth use (*χ*^2^_1_=19.5; *P*<.001) were significant covariates causing variance between paired sensitivity and specificity, whereas the operating system did not reveal such a difference (*χ*^2^_1_=0.02; *P*=.99). The AUC values for the elderly group versus the adult group were 0.90 (95% CI 0.87-0.92) versus 0.96 (95% CI 0.94-0.97), respectively, whereas the AUC values for the child group versus the adult group were 0.90 (95% CI 0.88-0.93) versus 0.96 (95% CI 0.94-0.97), respectively. The AUC values for the headphone group versus the earphone group were 0.96 (95% CI 0.94-0.97) versus 0.92 (95% CI 0.89-0.94), respectively. The AUC values for the soundproof booth group versus the non–soundproof booth group were 0.99 (95% CI 0.97-0.99) versus 0.94 (95% CI 0.91-0.96), respectively. The AUC values for the iPhone operating system group versus the Android operating system group were 0.95 (95% CI 0.93-0.97) versus 0.96 (95% CI 0.94-0.97), respectively. The detailed results are presented in Table 1.

**Table 1:** Results of the bivariate meta-regression analysis (N=25).

Covariate			Number		Sensitivity (95% CI)		*P* value		Specificity (95% CI)		*P* value		Likelihood-ratio test		Chi-square (*df*)		Area under the curve (95% CI)
**Age**																	
	Elderly [10,36,37,39]	4		0.77 (0.55-0.99)		.04		0.92 (0.80-1.00)		.99		<.001^a^		85.9 (1)		0.90 (0.87-0.92)	
	Child [21,22,38,41,49]	5		0.85 (0.69-1.00)		.10		0.96 (0.89-1.00)		.34		<.001		62.9(1)		0.90 (0.88-0.93)	
	Adult [29,40,42,44,46,48-52,54]	14		0.90 (0.85-0.96)		—^b^		0.91 (0.82-1.00)		—		—		—		0.96 (0.94-0.97)	
**Operating system**																	
	iPhone operating system [10,22,29,36,37,39,40,45,46,48,50,52,54]	17		0.88 (0.82-0.94)		.04		0.93 (0.87-0.99)		.51		.99		0.02(1)		0.95 (0.93-0.97)	
	Android [21,38,41,42,44,49,51]	8		0.89 (0.81-0.97)		—		0.93 (0.85-1.00)		—		—		—		0.96 (0.94-0.97)	
**Equipment**																	
	Headphone [10,21,38,41,42,44-47,49,50,52,54]	17		0.91 (0.87-0.95)		.85		0.89 (0.82-0.97)		.05		<.001		17.8(1)		0.96 (0.94-0.97)	
	Earphone [22,29,36,37,39,40,48]	7		0.80 (0.65-0.95)		—		0.97 (0.92-1.00)		—		—		—		0.92 (0.89-0.94)	
**Soundproof booth**																	
	Yes [10,49-51]	6		0.95 (0.90-1.00)		.72		0.95 (0.87-1.00)		.83		<.001		19.5(1)		0.99 (0.97-0.99)	
	No [21,22,29,36-42,44-46,52,54]	18		0.87 (0.82-0.93)		—		0.91 (0.85-0.98)		—		—		—		0.94 (0.91-0.96)	

^a^Significant *P*<.05.

^b^Reference of likelihood-ratio chi-square test.

### Publication Bias

**Figure 5:**
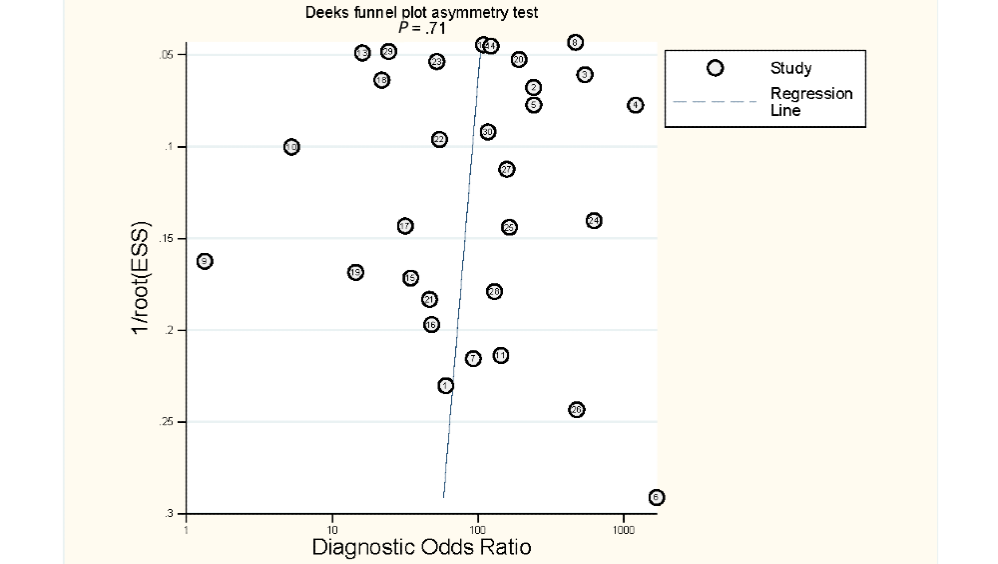
The Deeks funnel plot. ESS: effective sample size.

The Deeks funnel plot revealed no asymmetrical distribution for the included studies, and the regression test did not show a significant publication bias (*P*=.71; Figure 5).

## Discussion

### Principal Findings

In this study, we performed a meta-analysis to estimate the pooled diagnostic accuracy of smartphone-based hearing tests using conventional PTA as the gold standard. The overall sensitivity of smartphone-based audiometry was 89%, the specificity was 93%, and the AUC was 0.95, which suggested outstanding diagnostic performance for identifying hearing loss using PTA as the gold standard test. When using the SRT as the gold standard test, our results showed a sensitivity of 91%, specificity of 88%, and AUC of 0.93, which also indicated excellent diagnostic accuracy. On the basis of the results of the bivariate meta-regression analysis, we found that participant age, equipment used, and the use of a soundproof booth significantly affected the diagnostic accuracy of smartphone-based audiometry, whereas the operating system of the smartphone did not. To our knowledge, this is the first meta-analysis that provides comprehensive evidence of the diagnostic performance of a smartphone-based approach to detecting hearing loss.

PTA assesses a person’s lowest threshold response to pure tone stimuli at various frequencies [56]. It is still considered the gold standard test for audiologic examinations and provides information regarding the severity and type of hearing loss. According to the American National Standards Institute specifications, there are four types of PTA. Type 1 audiometry (advanced clinical or research) involves a completely equipped audiometer that can conduct both air and bone conduction tests. Type 2 (clinical) fits the same specifications as type 1, except for the requirement of loudspeaker equipment. Portable audiometers without speech-comprehension measurements are classified as type 3 (diagnostic), whereas type 4 (screening) consists of screening audiometers with the basic functions of a hearing test [56]. Although types 1 and 2 are considered the most informative and comprehensive audiometry, they are often not available in many settings, especially in resource-limited areas such as in low- and middle-income countries and rural regions. Even in resource-rich countries, standard PTA is not usually available at primary care practices [17]. Standard PTA tests require certified professionals to administer them, whereas audiologic training is generally lacking in resource-limited countries—there is less than one audiologist for every 1 million people according to previous studies [23,53,54]. Furthermore, the equipment for conventional PTA, including a soundproof booth and a calibrated audiometer, involves both cost and space. The demanding nature of conventional PTA may result in its low accessibility and further affect the generality of hearing screening and quality of hearing care [57,58].

In recent years, mobile health devices have evolved rapidly, as have smartphone-based hearing approaches. Smartphone-based audiometry is a cost-effective, convenient, and reliable tool for screening hearing loss. As smartphones are common in the modern society and the apps are very accessible, given their low cost or no cost, smartphone-based hearing tests could potentially bridge the gap between patients with hearing loss and adequate audiologic assessments and, potentially, hearing care. Previous studies have confirmed that such apps were able to provide basic hearing screening wherever the individual was located as long as the location met the required level of background noise, reducing the need to travel and pay for a hearing examination [59,60]. These smartphone-based hearing tests are usually designed to be user friendly because automated diagnostic audiometry simplifies complex audiologic protocols, allowing their use by nonprofessionals [61,62]**.** Studies have also described the use of smartphone-based audiometry in settings such as primary care practices and community health clinics for routine hearing screening to identify potentially handicapping hearing loss [59]. The findings of this study confirm that the diagnostic performance of smartphone-based audiometry aligns perfectly with conventional PTA in identifying hearing loss and adds to previous research with a larger pooled sample size and systematic scope.

Although this study highlights the high diagnostic value of smartphone-based hearing tests and their promising role in hearing screening, we identified several possible variables that may influence diagnostic performance. First, the accuracy of smartphone-based audiometry was lower in elderly individuals and children. This may suggest technical barriers between smart devices and elderly individuals and children. Previous studies have found that factors such as prevalent vision impairments and slower learning curves in managing technological devices because of lack of experience and functional decline may contribute to the higher level of difficulties when using smartphone-based apps among elderly populations [60,63]. At the same time, a previous study also found that children achieved lower accuracy in PTA [64]. Our results also showed that headphone use during the hearing examination may improve the diagnostic accuracy of smartphone-based audiometry. Earphones are a required component in standard audiometry because they prevent the collapse of the external ear canal and reduce the level of ambient noise [65-67]. However, if the participant does not insert the earphone correctly in the automated examination, it could be a problem. A previous study showed that earphone positioning may affect audiologic assessment results and whether the earphone is positioned by a trained examiner or by the examinee may affect audiologic assessment [68]. The negative effects of background noise may further support our finding that examinations conducted in soundproof booths have better diagnostic accuracy. The influence of ambient noise, which results in erroneous test results of smartphone audiometry, has been reported in previous studies [69,70], leading to the conclusion that the use of soundproof booths may increase the diagnostic accuracy of smartphone-based hearing tests [71]. Although some of the included studies reported comparable results of hearing assessments outside of a soundproof booth with passive attenuation and simultaneous ambient noise monitoring [71-73], most of the studies did not provide information regarding the management of ambient noise. The diagnostic value of this subgroup, however, still appeared feasible, because their AUC values exceeded the cutoff point of 0.9 [74]. In summary, our findings suggest that adequate adjustment of the variables that significantly affect the accuracy of smartphone-based audiometry may improve its diagnostic performance in diagnosing hearing loss. Approaches such as adding instructions regarding the examination protocol and correct use of earphones, providing customized audiologist consultations for elderly individuals, improving the app’s function in monitoring environmental noise, and regularly collecting feedback from users could be added to the current implementation methods.

### Limitations

This study has several limitations. First, as in most studies of diagnostic test accuracy, different thresholds exist among the studies and may have caused the threshold effect. A prior test calculation of the correlation between sensitivity and specificity revealed a negative result, confirming the threshold effect in this study. Therefore, we adopted the bivariate random-effects model to account for the cross-study threshold difference as suggested by previous studies [31,32]. Second, there was heterogeneity regarding the study designs, test protocols, and reference PTA thresholds for diagnosing hearing loss across the included studies, which may have biased the results when pooling them into the meta-analyses. Future studies with homogenous gold standards and uniform protocols for smartphone-based hearing tests are needed. Third, ambient noise monitoring is a key factor influencing the accuracy of audiometry [75]. Although most of the included studies did monitor noise, no data on the accuracy without ambient noise monitoring were provided. As a result, we were not able to perform the meta-regression analysis according to this factor. Fourth, frequency may act as a confounder, but most of the included studies did not provide diagnostic accuracy for each frequency; therefore, we could evaluate the diagnostic performance of smartphone audiometry only with the average threshold calculated from the frequencies. Fifth, most of the included studies did not describe the masking procedure, possibly because the included studies sampled healthy people, and the threshold difference between bilateral ears could hardly exceed 40 dB. In addition, some smartphone audiometry methods did not provide an automasking procedure during the automated examination. We suggest that future studies describe the masking procedure in detail, regardless of whether it is used. Sixth, of the 25 included studies, most did not describe the calibration method, whereas 9 (36%) used reference equivalent threshold sound pressure levels. A previous study revealed that the differences in hearing thresholds among the device models were significant, which might directly result from the biological calibration method used to determine the reference sound level [75]. Calibration information was lacking, possibly because of the intrinsic lack of a calibration function in the app. We suggest that future studies address this issue. Finally, some included studies enrolled patients with underlying diseases such as otitis media and SSNHL. Although, ideally, subgroup analyses should have been performed for these unique studies for more accurate results, we were not able to implement this investigation because of the scarcity of relevant studies. We look forward to more studies that investigate the value of smartphone audiometry in identifying different types of hearing loss in the future because they can provide more solid and specific evidence for apps in different clinical settings.

### Conclusions

In this meta-analysis, we have provided comprehensive evidence regarding the diagnostic performance of smartphone-based audiometry in diagnosing hearing loss. Given the high sensitivity and specificity of smartphone-based audiometry, along with its low cost and high accessibility, smartphone-based hearing assessments may serve as a cost-effective and equally accurate diagnostic tool, in comparison with conventional PTA, for assessing hearing loss, especially in resource-limited settings where conventional PTA is not feasible. Our findings also suggest that future improvements in smartphone-based audiometry should focus on adjusting the potential factors that may affect its diagnostic accuracy.

## Appendix

Multimedia Appendix 1 The detailed search strategy.

Multimedia Appendix 2 Table of study characteristics.

Multimedia Appendix 3 Table of study diagnostic parameters.

## Abbreviations

AUC: area under the receiver operating characteristic curve
ESS: effective sample size
PRISMA: Preferred Reporting Items for Systematic Reviews and Meta-Analyses
PTA: pure tone audiometry
SRT: speech recognition test
