# Supplementary material for: Diagnostic Accuracy of Smartphone-Based Audiometry for Hearing Loss Detection: Meta-analysis
Source: JMIR Mhealth Uhealth. 2021 Sep 10;9(9):e28378. doi: 10.2196/28378 (PMC8477297; doi:10.2196/28378)
Supplement: Multimedia Appendix 1 [file mhealth_v9i9e28378_app1.docx]

**Supplemental Table S1.** Detailed search strategy

| **Database** | **Query** |
| --- | --- |
| PubMed | ((( "Hearing Loss/analysis"[Mesh] OR "Hearing Loss/diagnosis"[Mesh] )) OR "Hearing Loss"[Mesh] OR "Hearing loss"[TIAB] OR "Hypoacusis"[TIAB] OR "Hearing Impairment"[TIAB] OR "Transitory Deafness"[TIAB] OR "Transitory Hearing Loss"[TIAB] OR "Hearing loss" OR "Hypoacusis" OR "Hearing Impairment" OR "Transitory Deafness" OR "Transitory Hearing Loss" ) AND (((("Cell Phone"[Mesh]) OR ( "Cell Phone/classification"[Mesh] OR "Cell Phone/instrumentation"[Mesh] OR "Cell Phone/statistics and numerical data"[Mesh] OR "Cell Phone/supply and distribution"[Mesh] )) OR "Smartphone"[Mesh]) OR ( "Smartphone/classification"[Mesh] OR "Smartphone/instrumentation"[Mesh] OR "Smartphone/statistics and numerical data"[Mesh] OR "Smartphone/supply and distribution"[Mesh] ) OR "Smartphones"[TIAB] OR "Smart Phones"[TIAB] OR "Smart Phone "[TIAB] OR "Cellular Phone"[TIAB] OR "Cellular Phones"[TIAB] OR "Cellular Telephone"[TIAB] OR "Cellular Telephones"[TIAB] OR "Cell Phones"[TIAB] OR "Portable Cellular Phone"[TIAB] OR "Portable Cellular Phones"[TIAB] OR "Mobile Phone"[TIAB] OR "Mobile Phones"[TIAB] OR "Mobile Telephone"[TIAB] OR "Mobile Telephones"[TIAB] OR "Car Phone"[TIAB] OR "Car Phones"[TIAB] OR "Mobile"[TIAB] OR "Smartphones" OR "Smart Phones" OR "Smart Phone " OR "Cellular Phone" OR "Cellular Phones" OR "Cellular Telephone" OR "Cellular Telephones" OR "Cell Phones" OR "Portable Cellular Phone" OR "Portable Cellular Phones" OR "Mobile Phone" OR "Mobile Phones" OR "Mobile Telephone" OR "Mobile Telephones" OR "Car Phone" OR "Car Phones" OR "Mobile") AND ((((((((("Diagnosis"[Mesh]) OR ( "Diagnosis/diagnosis"[Mesh] OR "Diagnosis/epidemiology"[Mesh] )) OR ( "Self-Examination/methods"[Mesh] OR "Self-Examination/statistics and numerical data"[Mesh] )) OR "Self-Examination"[Mesh]) OR "Self-Assessment"[Mesh]) OR "Audiometry"[Mesh]) OR ( "Audiometry/methods"[Mesh] OR "Audiometry/statistics and numerical data"[Mesh] )) OR ( "Hearing Tests/methods"[Mesh] OR "Hearing Tests/statistics and numerical data"[Mesh] )) OR "Hearing Tests"[Mesh] OR "Diagnosis"[TIAB] OR "Diagnoses"[TIAB] OR "Diagnose"[TIAB] OR "Diagnoses and Examinations"[TIAB] OR "Examinations and Diagnoses"[TIAB] OR "Self-Examinations"[TIAB] OR "Self Examination"[TIAB] OR "Self Examinations" OR "Assessment"[TIAB] OR "Self assessment"[TIAB] OR "Hearing test"[TIAB] OR "Hearing exam"[TIAB] OR "Audiometry"[TIAB] OR "Audiometric test"[TIAB] OR "Hearing screening"[TIAB] OR "Screening"[TIAB] OR "Audiometries"[TIAB] OR "Self-Assessments"[TIAB] OR "Diagnosis" OR "Diagnoses" OR "Diagnose" OR "Diagnoses and Examinations" OR "Examinations and Diagnoses" OR "Self-Examinations" OR "Self Examination" OR "Self Examinations" OR "Assessment" OR "Self assessment" OR "Hearing test" OR "Hearing exam" OR "Audiometry" OR "Audiometric test" OR "Hearing screening" OR "Screening" OR "Audiometries" OR "Self-Assessments") |
| Embase | ('hearing'/exp OR hypoacusis OR 'hearing impairment'/exp OR 'ransitory deafness' OR 'transitory hearing loss') AND (smartphones OR 'smart phones' OR 'smart phone' OR 'cellular phone' OR 'cellular phones' OR 'cellular telephone' OR 'cellular telephones' OR 'portable cellular phone' OR 'portable cellular phones' OR 'mobile phone'/exp OR 'mobile phones' OR 'mobile telephone' OR 'mobile telephones' OR 'car phone' OR 'car phones' OR mobile) AND ('diagnosis'/exp OR diagnoses OR diagnose OR 'diagnoses and examinations' OR 'examinations and diagnoses'/exp OR 'self examination'/exp OR 'self examinations' OR 'assessment'/exp OR 'self assessment' OR 'hearing test'/exp OR 'hearing exam' OR 'audiometry'/exp OR 'audiometric test' OR 'hearing screening' OR 'screening'/exp OR audiometries OR 'self assessments') |
| Web of Science | TS=(("Hearing loss" OR "Hypoacusis" OR "Hearing Impairment" OR "Transitory Deafness" OR "Transitory Hearing Loss")AND("Smartphones" OR "Smart Phones" OR "Smart Phone " OR "Cellular Phone" OR "Cellular Phones" OR "Cellular Telephone" OR "Cellular Telephones" OR "Cell Phones" OR "Portable Cellular Phone" OR "Portable Cellular Phones" OR "Mobile Phone" OR "Mobile Phones" OR "Mobile Telephone" OR "Mobile Telephones" OR "Car Phone" OR "Car Phones" OR "Mobile")AND("Diagnosis" OR "Diagnoses" OR "Diagnose" OR "Diagnoses and Examinations" OR "Examinations and Diagnoses" OR "Self-Examinations" OR "Self Examination" OR "Self Examinations" OR "Assessment" OR "Self assessment" OR "Hearing test" OR "Hearing exam" OR "Audiometry" OR "Audiometric test" OR "Hearing screening" OR "Screening" OR "Audiometries" OR "Self-Assessments")) |
| Scopus | (TITLE-ABS-KEY("Hearing loss" OR "Hypoacusis" OR "Hearing Impairment" OR "Transitory Deafness" OR "Transitory Hearing Loss") AND TITLE-ABS-KEY("Smartphones" OR "Smart Phones" OR "Smart Phone " OR "Cellular Phone" OR "Cellular Phones" OR "Cellular Telephone" OR "Cellular Telephones" OR "Cell Phones" OR "Portable Cellular Phone" OR "Portable Cellular Phones" OR "Mobile Phone" OR "Mobile Phones" OR "Mobile Telephone" OR "Mobile Telephones" OR "Car Phone" OR "Car Phones" OR "Mobile" ) AND TITLE-ABS-KEY("Diagnosis" OR "Diagnoses" OR "Diagnose" OR "Diagnoses and Examinations" OR "Examinations and Diagnoses" OR "Self-Examinations" OR "Self Examination" OR "Self Examinations" OR "Assessment" OR "Self assessment" OR "Hearing test" OR "Hearing exam" OR "Audiometry" OR "Audiometric test" OR "Hearing screening" OR "Screening" OR "Audiometries" OR "Self-Assessments")) |
| Cochrane Library | ID Search  #1 MeSH descriptor: [Hearing Loss] explode all trees  #2 MeSH descriptor: [Hearing Loss] explode all trees and with qualifier(s): [diagnosis - DI]  #3 Hearing loss  #4 Hypoacusis  #5 Hearing Impairment  #6 Transitory Deafness  #7 Transitory Hearing Loss  #8 {OR #1-#7}  #9 MeSH descriptor: [Smartphone] explode all trees  #10 MeSH descriptor: [Smartphone] explode all trees and with qualifier(s): [instrumentation - IS]  #11 MeSH descriptor: [Smartphone] explode all trees and with qualifier(s): [statistics & numerical data - SN]  #12 MeSH descriptor: [Smartphone] explode all trees and with qualifier(s): [classification - CL]  #13 MeSH descriptor: [Smartphone] explode all trees and with qualifier(s): [supply & distribution - SD]  #14 MeSH descriptor: [Cell Phone] explode all trees  #15 MeSH descriptor: [Cell Phone] explode all trees and with qualifier(s): [classification - CL]  #16 MeSH descriptor: [Cell Phone] explode all trees and with qualifier(s): [instrumentation - IS]  #17 MeSH descriptor: [Cell Phone] explode all trees and with qualifier(s): [statistics & numerical data - SN]  #18 MeSH descriptor: [Cell Phone] explode all trees and with qualifier(s): [supply & distribution - SD]  #19 Cellular Phone  #20 Cellular Phones  #21 Cellular Telephone  #22 Cellular Telephones  #23 Cell Phones  #24 Portable Cellular Phone  #25 Portable Cellular Phones  #26 Mobile Phone  #27 Mobile Phones  #28 Mobile  #29 Mobile Telephone  #30 Mobile Telephones  #31 Car Phone  #32 Car Phones  #33 Smartphones  #34 Smart Phones  #35 Smart Phone  #36 Smartphone  #37 {OR #9-#36}  #38 MeSH descriptor: [Diagnosis] explode all trees  #39 MeSH descriptor: [Self-Examination] explode all trees  #40 MeSH descriptor: [Self-Examination] explode all trees and with qualifier(s): [methods - MT]  #41 MeSH descriptor: [Self-Examination] explode all trees and with qualifier(s): [statistics & numerical data - SN]  #42 MeSH descriptor: [Self-Assessment] explode all trees  #43 MeSH descriptor: [Audiometry] explode all trees  #44 MeSH descriptor: [Audiometry] explode all trees and with qualifier(s): [methods - MT]  #45 MeSH descriptor: [Audiometry] explode all trees and with qualifier(s): [statistics & numerical data - SN]  #46 MeSH descriptor: [Hearing Tests] explode all trees  #47 MeSH descriptor: [Hearing Tests] explode all trees and with qualifier(s): [methods - MT]  #48 MeSH descriptor: [Hearing Tests] explode all trees and with qualifier(s): [statistics & numerical data - SN]  #49 Diagnosis  #50 Diagnoses  #51 Diagnose  #52 Diagnoses and Examinations  #53 Self-Examinations  #54 Self Examination  #55 Self Examinations  #56 Assessment  #57 Self assessment  #58 Hearing test  #59 Hearing exam  #60 Audiometry  #61 Audiometric test  #62 Hearing screening  #63 Screening  #64 Audiometries  #65 Self-Assessments  #66 {OR #38-#65}  #67 #8 AND #37 AND #66 |
