# Supplementary material for: Diagnostic Accuracy of Smartphone-Based Audiometry for Hearing Loss Detection: Meta-analysis
Source: JMIR Mhealth Uhealth. 2021 Sep 10;9(9):e28378. doi: 10.2196/28378 (PMC8477297; doi:10.2196/28378)
Supplement: Multimedia Appendix 2 [file mhealth_v9i9e28378_app2.docx]

**Multimedia Appendix 2.** Table of study characteristics (N=25).

| Study | Country | Age (years), mean | App | Sex | Operating system | Equipment | Noise monitoring (dB) | Masking (threshold difference, dB) | Calibration | Soundproof booth | Modality |
| --- | --- | --- | --- | --- | --- | --- | --- | --- | --- | --- | --- |
| Abu-Ghanem et al [36] | Israel | 84.4 | uHear | Male: 8; female: 18 | iOS | Earphone | <50 | NR^c^ | NR | No | PTA^d^ |
| Anuar et al [48] | Malaysia | 33 | uHear | Male: 73; female: 67 | iOS | Earphone | NR | NR | NR | NR | PTA |
| Aremu et al [51] | Nigeria | 45 | Hearing Test Pro | Male: 100; female: 80 | Android | NR | NR | NR | NR | Yes | PTA |
| Bauer et al [45] | Brazil | NR | Test 1: HearCheck; test 2: Ouviu | Male: 73; female: 112 | iOS | Headphone | <40 | NR | NR | No | PTA |
| Chu et al [22] | Taiwan | 11 | Ear Scale | Male: 38; female: 47 | iOS | Earphone | <50 | NR | RETSPLs^c^ | No | PTA |
| Corona et al [49] | Brazil | Adult: 53; child: 9 | hearTest | NR | Android | Headphone | <44 | NR | NR | Yes | PTA |
| Derin et al [54] | Turkey | 53.6 | EarTrumpet | NR | iOS | Headphone | <35 | >35 | NR | No | PTA |
| Durgut et al [38] | Turkey | 8.2 | Hearing Test | Male: 26; female: 24 | Android | Headphone | <40 | NR | NR | No | PTA |
| Handzel et al [29] | United States | 51.4 | uHear | Male: 24; female: 8 | iOS | Earphone | <4 | NR | NR | No | PTA |
| Kelly et al [46] | United States | Group 1: 61  Group 2: 63  Group 3: 45 | Group 1: EarTrumpet; group 2: Audiogram; group 3: Hearing Test | Group 1: male: 16; female: 19  Group 2: male: 26; female: 11  Group 3: male: 20; female: 15 | iOS | Headphone | <70 | NR | RETSPLs | No | PTA |
| Li et al [10] | Taiwan | 72.3 | uHear | Male: 27; female: 14 | iOS | Headphone | <35 | NR | NR | Yes | PTA |
| Lin et al [52] | Taiwan | 46 | Ear Scale | Male: 44; female: 44 | iOS | Headphone | <50 | NR | RETSPLs | No | PTA |
| Livshirz et al [37] | Israel | 74.6 | uHear | Male: 28; female: 32 | iOS | Earphone | <35 | NR | NR | No | PTA |
| Louw et al [42] | South Africa | 48.5 | hearScreen | Male: 66; female: 183 | Android | Headphone | <35 | >75 | RETSPLs | No | PTA |
| Lycke et al [39] | Belgium | 76.4 | uHear | Male: 24; female: 21 | iOS | Earphone | <50 | NR | NR | No | PTA |
| Mahomed-Asmail et al [41] | South Africa | 8 | hearScreen | Male: 528; female: 542 | Android | Headphone | <25 | NR | RETSPLs | No | PTA |
| Peer et al [40] | South Africa | 43 | uHear | Male: 13; female: 12 | iOS | Earphone | <40 | NR | NR | No | PTA |
| Potgieter et al [43] | South Africa | 36 | DIN^d^ | Male: 164; female: 290 | Android | Headphone | NR | NR | NR | No | SRT^e^ |
| Potgieter et al [53] | South Africa | 55 | DIN | Male: 43; female: 66 | Android | Earphone | NR | NR | NR | yes | SRT |
| Saliba et al [50] | Canada | 49.7 | Group 1: EarTrumpet; group 2: ShoeBOX | Male: 19; female: 14 | iOS | Headphone | <50 | >35 | RETSPLs | Yes | PTA |
| Samelli et al [47] | Brazil | 22.3 | Self-designed | Male: 6; female: 24 | iOS | Headphone | <50 | NR | RETSPLs | No | SRT |
| Sandstrom et al [44] | Sweden | 52 | hearTest | NR | Android | Headphone | NR | NR | RETSPLs | No | PTA |
| Swanepoel et al [21] | South Africa | 6.5 | hearScreen | NR | Android | Headphone | NR | NR | RETSPLs | No | PTA |
| Xiao et al [55] | United States | 6.5 | Kids Hearing Game | Male: 326; female: 190 | iOS | Earphone | <20 | >35 | NR | Yes | S  RT |
| Yimtae et al [23] | Thailand | 4.7 | Screening | NR | Android | Headphone | <20 | NR | NR | No | SRT |

^a^NR: not reported.

^b^PTA: pure tone audiometry.

^c^RETSPL: reference equivalent threshold sound pressure levels.

^d^DIN: digits-in-noise test.

^e^SRT: speech recognition test.
