# Supplementary material for: Diagnostic Accuracy of Smartphone-Based Audiometry for Hearing Loss Detection: Meta-analysis
Source: JMIR Mhealth Uhealth. 2021 Sep 10;9(9):e28378. doi: 10.2196/28378 (PMC8477297; doi:10.2196/28378)
Supplement: Multimedia Appendix 3 [file mhealth_v9i9e28378_app3.docx]

**Multimedia Appendix 3.** Table of study diagnostic parameters (N=25).

| Study | Threshold (dB) | Frequency (kHz) | True positive | False positive | False negative | True negative | Prevalence | Sensitivity (95% CI) | Specificity (95% CI) |
| --- | --- | --- | --- | --- | --- | --- | --- | --- | --- |
| Abu-Ghanem et al [36] | 25 | 0.25, 0.5, 1, 2, 4, 6 | 3 | 0 | 2 | 21 | 0.808 | 0.60 (0.15-0.95) | 1.00 (0.84-1.00) |
| Anuar et al [48] | 40 | 0.25, 0.5, 1, 2, 4, 8 | 40 | 1 | 34 | 205 | 0.264 | 0.54 (0.42-0.66) | 1.00 (0.97-1.00) |
| Aremu et al [51] | 25 | 0.25, 0.5, 1, 2, 4, 8 | 204 | 0 | 67 | 89 | 0.753 | 0.75 (0.70-0.80) | 1.00 (0.96-1.00) |
| Bauer et al [45] | 20 (1, 2, 4 kHz) +40 (0.5, 8 kHz) | 0.5, 1, 2, 4, 8 | 96 | 1 | 25 | 63 | 0.651 | 0.98 (0.93-0.99) | 0.97 (0.89-1.00) |
| Bauer et al [45] | 20 (1, 2, 4 kHz) +40 (0.5, 8 kHz) | 0.5, 1, 2, 4, 8 | 117 | 2 | 3 | 62 | 0.651 | 0.79 (0.71-0.86) | 0.98 (0.92-1.00) |
| Chu et al [22] | 25 | 0.5, 1, 2, 4 | 2 | 0 | 0 | 168 | 0.012 | 1.00 (0.16-1.00) | 1.00 (0.98-1.00) |
| Corona et al [49] | 25 | 0.5, 1, 2, 4 | 234 | 62 | 2 | 248 | 0.432 | 1.00 (0.48-1.00) | 0.90 (0.79-0.97) |
| Corona et al [49] | 25 | 0.5, 1, 2, 4 | 5 | 5 | 0 | 46 | 0.089 | 0.99 (0.97-1.00) | 0.80 (0.75-0.84) |
| Derin et al [54] | 20 | 0.5, 1, 2, 4 | 36 | 9 | 9 | 3 | 0.813 | 0.80 (0.65-0.90) | 0.25 (0.05-0.57) |
| Durgut et al [38] | 20 | 0.5, 1, 2, 4 | 44 | 39 | 3 | 14 | 0.470 | 0.94 (0.82-0.99) | 0.26 (0.15-0.40) |
| Handzel et al [29] | 40 | 0.5, 1, 2, 4, 6 | 24 | 1 | 1 | 6 | 0.781 | 0.96 (0.80-1.00) | 0.86 (0.42-1.00) |
| Kelly et al (EarTrumpet) [46] | 20 | 0.5, 1, 2, 4 | 252 | 39 | 10 | 190 | 0.534 | 0.85 (0.81-0.89) | 0.95 (0.91-0.97) |
| Kelly et al (Audiogram) [46] | 20 | 0.5, 1, 2, 4 | 254 | 11 | 44 | 208 | 0.577 | 0.88 (0.82-0.92) | 0.69 (0.63-0.75) |
| Kelly et al (Hearing Test) [46] | 20 | 0.5, 1, 2, 4 | 170 | 69 | 24 | 157 | 0.461 | 0.96 (0.93-0.98) | 0.83 (0.77-0.88) |
| Li et al [10] | 40 | 0.5, 1, 2, 4 | 11 | 7 | 1 | 22 | 0.293 | 0.92 (0.62-1.00) | 0.76 (0.56-0.90) |
| Lin et al [52] | 25 | 0.5, 1, 2, 4 | 77 | 2 | 4 | 5 | 0.920 | 0.95 (0.88-0.99) | 0.71 (0.29-0.96) |
| Livshirz et al [37] | 35 | 1, 2, 4 | 13 | 4 | 4 | 39 | 0.283 | 0.76 (0.50-0.93) | 0.91 (0.78-0.97) |
| Louw et al [42] | 25 | 1, 2, 4 | 113 | 19 | 25 | 92 | 0.554 | 0.82 (0.74-0.88) | 0.83 (0.75-0.89) |
| Lycke et al [39] | 40 | 0.5, 1, 2 | 8 | 4 | 4 | 29 | 0.267 | 0.67 (0.35-0.90) | 0.88 (0.72-0.97) |
| Mahomed-Asmail et al [41] | 25 | 1, 2, 4 | 75 | 15 | 25 | 956 | 0.093 | 0.75 (0.65-0.83) | 0.98 (0.97-0.99) |
| Peer et al [40] | 40 | 0.5, 1, 2, 4 | 8 | 11 | 0 | 31 | 0.720 | 1.00 (0.63-1.00) | 0.74 (0.58-0.86) |
| Potgieter et al [43] | 25 | 0.5, 1, 2, 4 | 110 | 78 | 7 | 259 | 0.258 | 0.88 (0.76-0.95) | 0.88 (0.77-0.95) |
| Potgieter et al [53] | 25 | 0.5, 1, 2, 4 | 44 | 7 | 6 | 52 | 0.459 | 0.94 (0.88-0.98) | 0.77 (0.72-0.81) |
| Saliba et al [50] | 40 | 0.5, 1, 2, 4, 8 | 14 | 2 | 2 | 47 | 0.246 | 1.00 (0.79-1.00) | 0.96 (0.86-1.00) |
| Saliba et al [50] | 40 | 0.5, 1, 2, 4, 8 | 16 | 2 | 0 | 47 | 0.246 | 0.88 (0.62-0.98) | 0.96 (0.86-1.00) |
| Samelli et al [47] | 20 (1, 2, 4 kHz) +30 (0.5 kHz) | 0.5, 1, 2, 4 | 4 | 0 | 0 | 26 | 0.133 | 1.00 (0.40-1.00) | 1.00 (0.87-1.00) |
| Sandstrom et al [44] | 40 | 0.5, 1, 2, 4 | 29 | 3 | 3 | 49 | 0.381 | 0.91 (0.75-0.98) | 0.94 (0.84-0.99) |
| Swanepoel et al [21] | 25 | 1, 2, 4 | 5 | 4 | 3 | 312 | 0.025 | 0.63 (0.24-0.91) | 0.99 (0.97-1.00) |
| Xiao et al [55] | 20 | 0.5, 1, 2, 4, 8 | 139 | 105 | 14 | 258 | 0.297 | 0.91 (0.85-0.95) | 0.71 (0.66-0.76) |
| Yimtae et al [23] | 25 | 0.5, 1, 2, 4 | 34 | 9 | 4 | 124 | 0.222 | 0.89 (0.75-0.97) | 0.93 (0.88-0.97) |
